# Nomogram model for predicting invasive placenta in patients with placenta previa: integrating MRI findings and clinical characteristics

**DOI:** 10.1038/s41598-023-50900-z

**Published:** 2024-01-02

**Authors:** Fang Huang, Guo-Rong Lyu, Qing-Quan Lai, Yuan-Zhe Li

**Affiliations:** 1 Department of Radiology, The Second Affiliated Hospital of Fujian Medical University, Quanzhou, China; 2 Department of Radiology, The Second Affiliated Clinical Medical College of Fujian Medical University, Quanzhou, China; 3 Department of Ultrasound, The Second Affiliated Hospital of Fujian Medical University, Quanzhou, China; 4 Department of Ultrasound, The Second Affiliated Clinical Medical College of Fujian Medical University, Quanzhou, China

**Keywords:** Reproductive disorders, Diagnosis

## Abstract

This study aims to validate a nomogram model that predicts invasive placenta in patients with placenta previa, utilizing MRI findings and clinical characteristics. A retrospective analysis was conducted on a training cohort of 269 patients from the Second Affiliated Hospital of Fujian Medical University and a validation cohort of 41 patients from Quanzhou Children’s Hospital. Patients were classified into noninvasive and invasive placenta groups based on pathological reports and intraoperative findings. Three clinical characteristics and eight MRI signs were collected and analyzed to identify risk factors and develop the nomogram model. The mode’s performance was evaluated in terms of its discrimination, calibration, and clinical utility. Independent risk factors incorporated into the nomogram included the number of previous cesarean sections ≥ 2 (odds ratio [OR] 3.32; 95% confidence interval [CI] 1.28–8.59), type-II placental bulge (OR 17.54; 95% CI 3.53–87.17), placenta covering the scar (OR 2.92; CI 1.23–6.96), and placental protrusion sign (OR 4.01; CI 1.06–15.18). The area under the curve (AUC) was 0.908 for the training cohort and 0.803 for external validation. The study successfully developed a highly accurate nomogram model for predicting invasive placenta in placenta previa cases, based on MRI signs and clinical characteristics.

## Introduction

The placenta accreta spectrum (PAS) encompasses abnormal adhesion of the placental trophoblast to the uterine myometrium^1^. The Federation of International of Gynecologists and Obstetricians (FIGO) classified PAS in 2018 into three grades: Grade 1 (placenta accreta), where the placenta attaches directly to the myometrium surface without invasion; Grade 2 (placenta increta), involving invasion into the myometrium; and Grade 3 (placenta percreta), where invasion extends to surrounding pelvic tissues, vessels, and organs^2^. PAS Grades 2 and 3 are categorized as invasive placenta, while non-adherent placenta and PAS Grade 1 are considered noninvasive^3^. PAS, particularly during delivery, can lead to massive intrapartum or postpartum hemorrhages, posing risks of hysterectomy or maternal mortality^4–6^. Early prenatal detection of PAS is crucial for reducing maternal morbidity by facilitating advance treatment planning^7,8^.

Recent studies have focused on MRI signs for PAS prediction, exploring their potential in diagnostic models. Some researchers have proposed prediction models integrating MRI findings and clinical characteristics, quantifying MRI signs into a scoring system. Ueno et al. developed the first MRI-based scoring system for PAS in 2016, utilizing a Likert scale to score six MRI signs, demonstrating excellent diagnostic performance^9^. Delli and colleagues also created a new prediction model for PAS and clinical outcomes based on MRI signs, identifying abnormal vascularity and percretism signs as the most predictive features of PAS^10^. Similarly, Li’s team developed a nomogram using MRI to predict the invasive form of PAS disorder^11^. However, these studies were limited by small sample sizes or lacked external validation. Thus, our study aims to develop and validate a comprehensive PAS prediction model based on both MRI signs and clinical characteristics.

## Materials and methods

### Research subjects

This study received approval from the institutional review boards of both the Second Affiliated Hospital of Fujian Medical University and Quanzhou Children’s Hospital (Reference No. 463). Due to its retrospective nature, written informed consent was waived, as approved by the Ethics Committees of the two hospitals. The study adhered to the guidelines of the Declaration of Helsinki and was conducted in compliance with relevant guidelines and regulations.

The sample size for our clinical prediction model was determined using Riley’s methods, employing the “pmsampsize” package in R^12^. Based on our prior clinical observations, the incidence rate of PAS ranges between 20 and 25%. Consequently, we adopted a midpoint rate of 22% for our calculations. This approach indicated a minimum required sample size of 264, assuming the inclusion of five predictive factors. Additionally, in developing the model, we adhered to the guideline of ensuring a minimum of 10 events per predictive factor.

**Figure 1:**
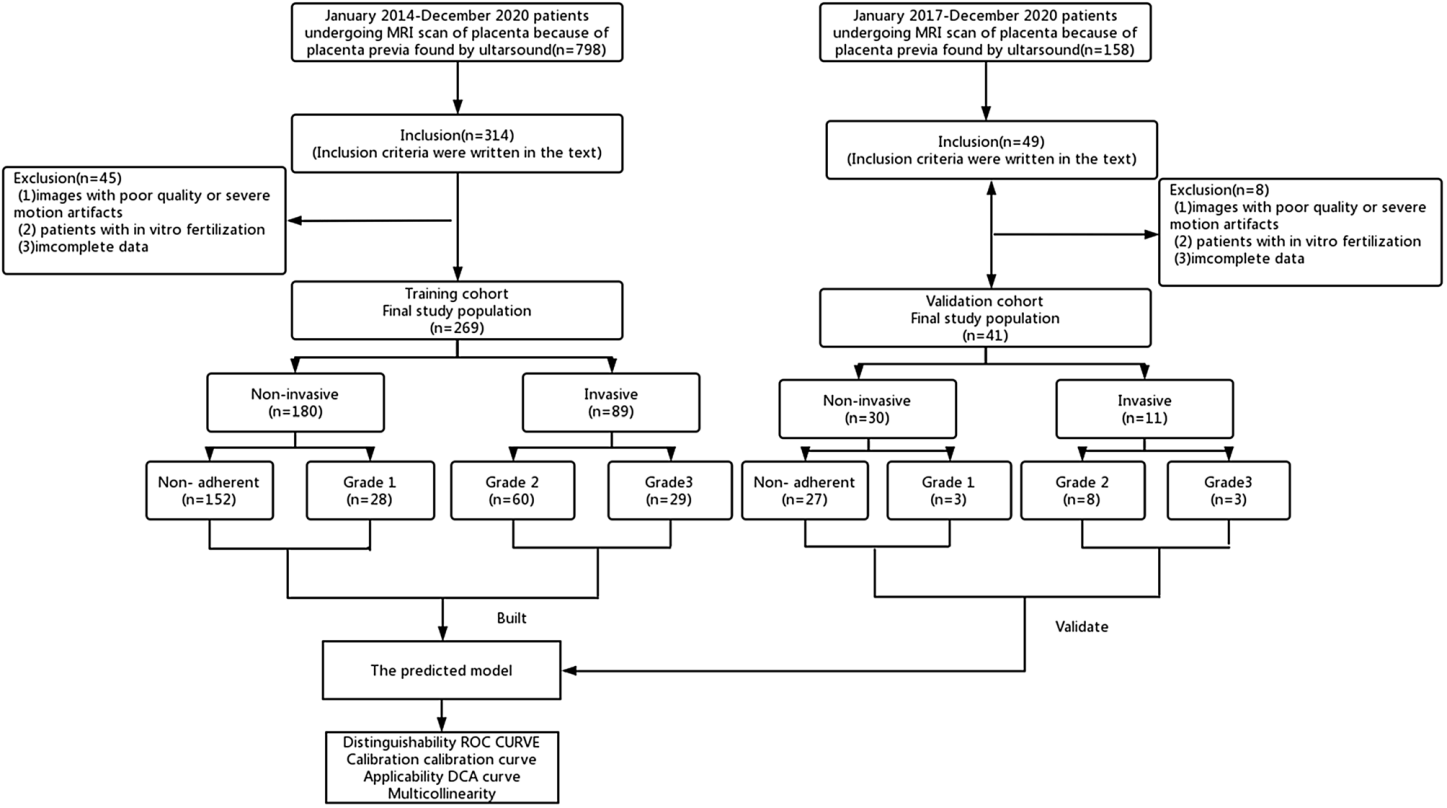
Flowchart of participants.

Placenta previa is increasingly recognized as a risk factor for PAS. Patients suspected of placenta previa, identified via ultrasound, are typically recommended for further MRI scans to exclude PAS^13,14^. From January 2014 to December 2020, we included 798 patients from the Second Affiliated Hospital of Fujian Medical University who underwent MRI scans for placenta previa as identified by ultrasound. The inclusion criteria were: (1) placenta previa confirmed by MRI after 28 weeks of gestation; (2) prenatal ultrasound and MRI conducted in our hospital; (3) singleton pregnancies; (4) cesarean section deliveries and any related surgeries performed in our hospital; (5) absence of prior hysteroscopic surgery; (6) the last MRI assessment of the placenta conducted within two weeks before cesarean section. Exclusion criteria were: (1) poor quality images or severe motion artifacts; (2) patients with in vitro fertilization due to the potential higher risk of invasive placenta; (3) incomplete data. Ultimately, 269 patients were included as a training cohort, with relevant clinical and radiological data retrospectively collected. Similarly, 158 patients from Quanzhou Children’s Hospital during the same period were considered, with 41 patients eventually enrolled as a validation cohort. Clinical and radiological data were collected and compared between the cohorts. The process is illustrated in Fig. 1.

### Instruments and equipment

MRI scans were performed using a 1.5 T MR scanner (Achieva, Philips Medical System, Netherlands) equipped with a body array coil covering the entire pelvis. To facilitate optimal evaluation of the bladder-serosal interface, patients were instructed to maintain a full bladder during the scan. The scanning sequences included ssh_TSE, BTFE DWI, and T1WI. The BTFE parameters were TR 3.6 ms, TE 2.0 ms, with a slice thickness of 5 mm, interslice spacing of 0.6 mm, field of view (FOV) 36 cm × 36 cm, and a matrix size of 320 × 270. The ssh_TSE parameters were TR 800 ms, TE 87 ms, slice thickness 5 mm, interslice spacing 0.6 mm, FOV 36 cm × 36 cm, and a matrix size of 384 × 256. T1WI parameters included TR 4.27 ms, TE 1.56 ms, slice thickness 6 mm, interslice spacing 1 mm, FOV 36 cm × 36 cm, and a matrix size of 256 × 192. DWI settings (b values = 0,800 s/mm2) were TR 400 ms, TE 75 ms, slice thickness 5 mm, interslice spacing 0.6 mm, FOV 36 cm × 36 cm, and a matrix size of 256 × 256. Images were acquired in three planes: coronal, sagittal, and transverse. The application of SENSE technology facilitated shorter scanning times, reduced motion artifacts, and enhanced image quality. No gadolinium-based contrast agents were administered during the study.

### MRI image evaluation

**Figure 2:**
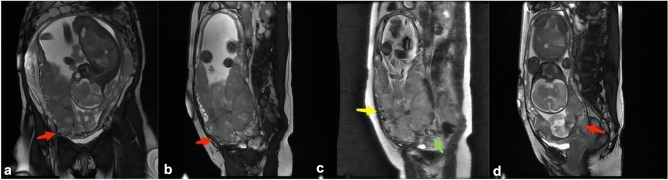
39 yrs old, PAS grade 3. A and B: the red arrow show the sign of placental bulge type-II on the OCOR (**a**) and OSAG (**b**) BTFE sequence; (**c**): the yellow arrow show the sign of uterine serosal hypervascularity, the green arrow show sign of bridging vessels on OSAG ssh_TSE sequence; (**d**): the red arrow show the sign of placental protrusion on OSAG BTFE sequence.

**Figure 3:**
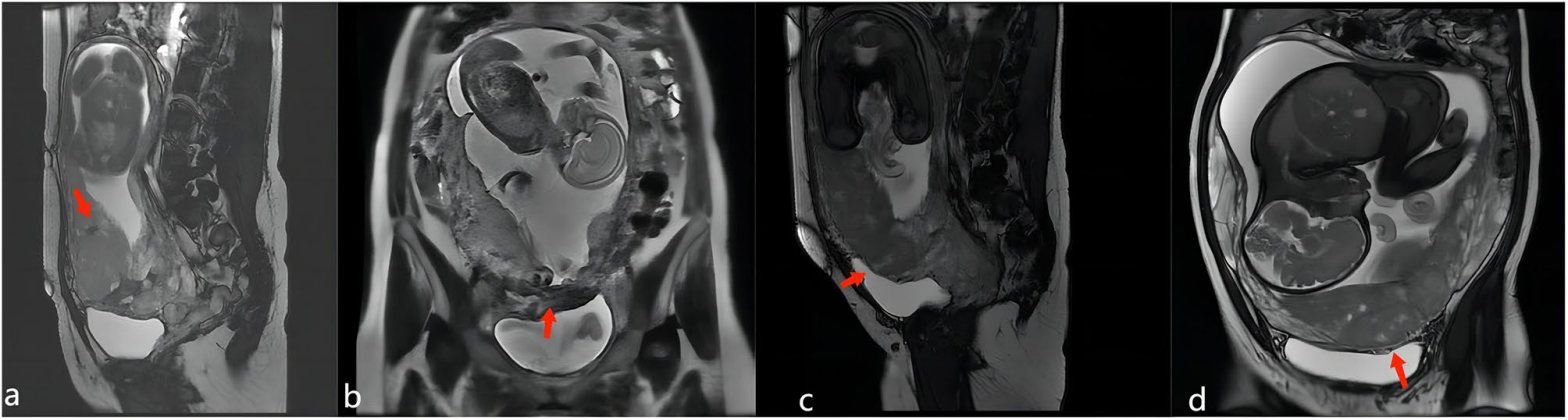
A and B are 34 yrs old, PAS grade 3. A:the red arrow show the sign of intraplacental T2 dark bands on the OSAG BTFE sequence; B: the red arrow show the sign of placental recess on the OCOR ssh_TSE sequence; (**c**) and (**d**) are 29 yrs old, PAS grade 2. The red arrow show the sign of placental bulge type-I on the OSAG (**a**) and OCOR (**b**) BTFE sequence.

The MRI images were reviewed by two experienced radiologists, with 10 and 15 years of experience in pelvic MRI diagnostics, respectively. Prior to the study, these radiologists underwent training to standardize the interpretation of MRI images. They were blinded to patients’ clinical histories, previous radiological reports, and surgical and pathological outcomes. In cases of disagreement, a consensus was reached by accepting the positive diagnosis made by either radiologist. Both radiologists contributed equally to the data used in constructing the nomogram model. Key MRI features indicative of abnormal placentation, as identified in the literature, were assessed and included: (1) Intraplacental T2 dark bands; (2) Placental bulge type-I; (3) Placental bulge type-II; (4) Uterine serosal hypervascularity; (5) Abnormal intraplacental vascularity and/or bridging vessels; (6) Placental recess; (7) Placenta covering scar; (8) Placental protrusion sign^15,16^. These signs are detailed in Table 1, with examples illustrated in Figs. 2 and 3. Additionally, patient age, number of abortions and cesarean sections, gestational age at MRI, and gestational age at delivery were recorded.

**Table 1:** The definition of MR signs.

MR signs	Definition
Intraplacental T2 dark bands	Nodular or linear areas of low signal intensity > 2 cm
Placental bulge type-I	A focal bulge slightly outwards into the myometrium but the outline is not distorted
Placental bulge type-II	Outline shape is distorted
Uterine serosal hypervascularity	Tortuous flow voids along the uterine serosa
Abnormal intraplacental vascularity and/or bridging vessels	Enlarged and tortuous vessels with a diameter > 6 mm within the placenta. Bridging vessels is the vessel running perpendicular to the placenta and serosal layer
Placental recess	Wedge-shaped contraction of the placental surface and uterine outer rim accompanying a T2 dark band
Placenta covering scar	The placenta cover the scar of previous cesarean section
Placental protrusion sign	The placenta extend into the internal uterine

### PAS grading and patient classification

According to the FIGO, the PAS grading diagnosis is confirmed by pathological reports and intrapartum findings^17^. Patients were categorized into noninvasive placenta groups (including PAS grade 1 and non-adherent placenta) and invasive placenta groups (including PAS grades 2 and 3). The diagnostic criteria and classification of noninvasive and invasive placenta are outlined in Table 2. All patients in this study underwent cesarean section delivery, with PAS grading determined through a combination of intraoperative findings and pathological results. All suspected cases of placental implantation were routinely referred for pathological analysis. The interpretation of intraoperative findings and pathological diagnoses was overseen by an obstetrician with over 20 years of experience, while the pathological examination was conducted by a pathologist with more than 15 years of experience.

**Table 2:** Diagnostic criteria and classification of noninvasive placenta and invasive placenta.

	Intraoperative findings	Pathological results
Non-adherent	The placenta can be peeled off on its own	
Grade 1	The placenta cannot be peeled off on its own, it needs to be peeled off by hand. The placental detach surface is rough and bleeding after stripping	The placental villi are attached to the myometrium
Grade 2	Abnormal dilated placental blood vessels are seen during the operation. The placenta cannot be detached by itself and the placenta is torn by hand, which required oval forceps to remove or cut off the implanted part of the myometrium	The villi can be seen invading the myometrium
Grade 3	The uterine surface is purple-blue during the operation, and the vascular network abundantly distended or penetrated the uterine serosa to the adjacent organs	The local myometrial structure disappears and is replaced by placental tissue

### Statistical analysis

Statistical analyses were performed using SPSS version 23.0 (SPSS, Inc, Chicago, IL, USA) and R software (R version 4.0.3, The R Project for Statistical Computing, www.r-project.org). Continuous variables were presented as mean ± standard deviation, while categorical variables were expressed as numbers (percentages). The Chi-square test was employed for analyzing categorical variables. Multivariate logistic regression analysis was conducted using significant risk factors identified. The variance inflation factor (VIF) was calculated to assess collinearity, with a VIF < 5 indicating no significant collinearity. A *p* value < 0.05 was considered statistically significant.

A nomogram was created using the coefficients from the logistic regression model, weighted in the R package “rms”. The discriminative performance of the model was assessed by the area under the receiver operating characteristic (ROC) curve. Calibration was evaluated using unreliability statistics to generate a calibration curve. Bootstrapping with 1000 samples was performed to assess internal calibration and discrimination. The clinical utility of the nomogram was evaluated using decision curve analysis (DCA), determining the net benefit at various threshold probabilities.

## Results

### Clinical and radiological variables analysis

The clinical and radiological characteristics of the patients are summarized in Table 3. In the training cohort of 269 patients, 180 were classified into the noninvasive group and 89 into the invasive group. The distribution was as follows: 152 patients (56.5%) were nonadherent, 28 (10.4%) were PAS grade 1, 60 (22.3%) were PAS grade 2, and 29 (10.8%) were PAS grade 3. No significant differences were observed in age and number of abortions between the groups. However, significant differences were found in the number of previous cesarean sections ≥ 2 and various MRI signs, including intraplacental T2 dark bands, placental bulge type I, placental bulge type II, uterine serosal hypervascularity, abnormal intraplacental vascularity and/or bridging vessels, placental recess, placenta covering scar, and placental protrusion sign (Table 3). Multivariable logistic regression analysis identified the number of previous cesarean sections ≥ 2, placental bulge type-II, placenta covering scar, and placental protrusion sign as independent risk factors for invasive placenta (Table 4). The analysis indicated increased risks for invasive placenta by factors of 3.32, 17.57, 2.94, and 4.01, respectively, for these variables. No significant collinearity was found among the significant predictors (Table 5).

**Table 3:** The clinical and radiological variables of patients of training cohort.

Variable	Noninvasive (180)	Invasive (89)	χ^2^ value	*P* value
Age (years)				
< 35	130 (72.2%)	58 (65.2%)	1.408	0.235
≥ 35	50 (27.8%)	31 (34.8%)		
Number of previous cesarean section				
< 2	165 (91.7%)	48 (53.9%)	51.442	< 0.001
≥ 2	15 (8.3%)	41 (46.1%)		
Number of previous abortion				
< 2	102 (56.7%)	46 (51.7%)	0.597	0.440
≥ 2	78 (43.3%)	43 (48.3%)		
Intraplacental T2 dark bands				
Negative	165 (91.7%)	30 (33.7%)	100.320	< 0.001
Positive	15 (8.3%)	59 (66.3%)		
Placental bulge type-I				
Negative	126 (70.0%)	50 (56.2%)	5.029	0.025
Positive	54 (30.0%)	39 (43.8%)		
Placental bulge type-II				
Negative	177 (98.3%)	38 (42.7%)	114.896	< 0.001
Positive	3 (1.7%)	51 (57.3%)		
Uterine serosal hypervascularity				
Negative	158 (87.8%)	36 (40.4%)	66.343	< 0.001
Positive	22 (12.2%)	53 (59.6%)		
Abnormal intraplacental vascularity and/or bridging vesselss				
Negative	153 (85.0%)	39 (43.8%)	83.655	< 0.001
Positive	27 (15.0%)	50 (56.2%)		
Placental recess				
Negative	175 (97.2%)	53 (59.6%)	65.422	< 0.001
Positive	5 (2.8%)	36 (40.4%)		
Placenta covering scar				
Negative	135 (75.0%)	15 (16.9%)	81.624	< 0.001
Positive	45 (25.0%)	74 (83.1%)		
Placental protrusion sign				
Negative	176 (97.8%)	49 (55.1%)	79.446	< 0.001
Positive	4 (2.2%)	40 (44.9%)

**Table 4:** Significant predictors of the invasive placenta from multivariate logistic regression analysis.

Variable	OR(95% CI value)	*P* value
Number of previous cesarean sections ≥ 2	3.32(1.28–8.59)	0.013
Placental bulge type-II	17.54(3.53–87.17)	0.000
Placenta covering scar	2.92(1.23–6.96)	0.015
Placental protrusion sign	4.01(1.06–15.18)	0.041

**Table 5:** Collinearity of significant predictors of invasive placenta.

Variable	VIF
Number of previous cesarean sections ≥ 2	1.26
Placental bulge type-II	1.97
Placenta covering scar	1.56
Placental protrusion sign	1.70

### Validation cohort

The validation cohort from Quanzhou Children’s Hospital comprised 41 patients. There were no significant differences in relevant variables between the two cohorts (Table 6).

**Table 6:** Comparison of significant predictors between the training and validation cohorts.

Variable	Training cohort (269)	Validation cohort (41)	χ^2^ value	*P* value
Number of previous cesarean sections				
< 2	213	32	0.028	0.867
≥ 2	56	9		
Placental bulge type-II				
Negative	215	35	0.675	0.411
Positive	54	6		
Placenta covering scar				
Negative	150	26	0.849	0.357
Positive	119	15		
Placental protrusion sign				
Negative	225	38	2.26	0.132
Positive	44	3		

### Nomogram model creation and evaluation

**Figure 4:**
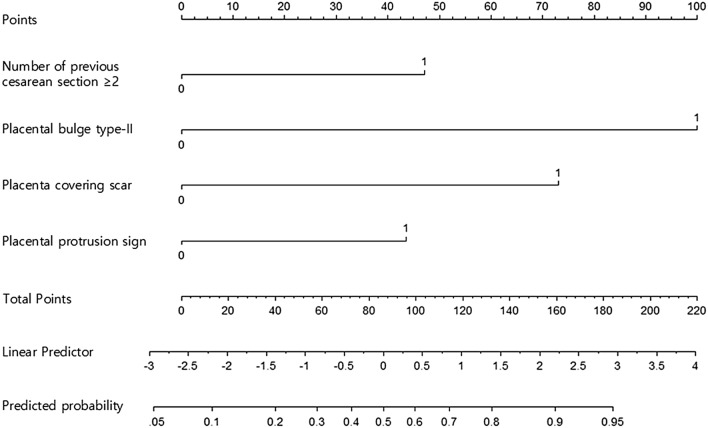
Nomogram model for predicting invasive placenta. For a given patient, use the axis at the top of the figure and points are assigned to each variable and get the total score. The total score corresponds to a predicted probability of invasive placenta.

Utilizing the coefficients from the final model, a nomogram was developed (Fig. 4). The first line on the nomogram provides reference scores for each risk factor. Lines 2–5 represent the scores assigned to each individual risk factor, including the number of previous cesarean sections ≥ 2, placental bulge type-II, placenta covering scar, and placental protrusion sign. The sixth line denotes the total score, calculated by summing the scores of each risk factor. The final line on the nomogram represents the predicted probability of invasiveness as determined by the model.

**Figure 5:**
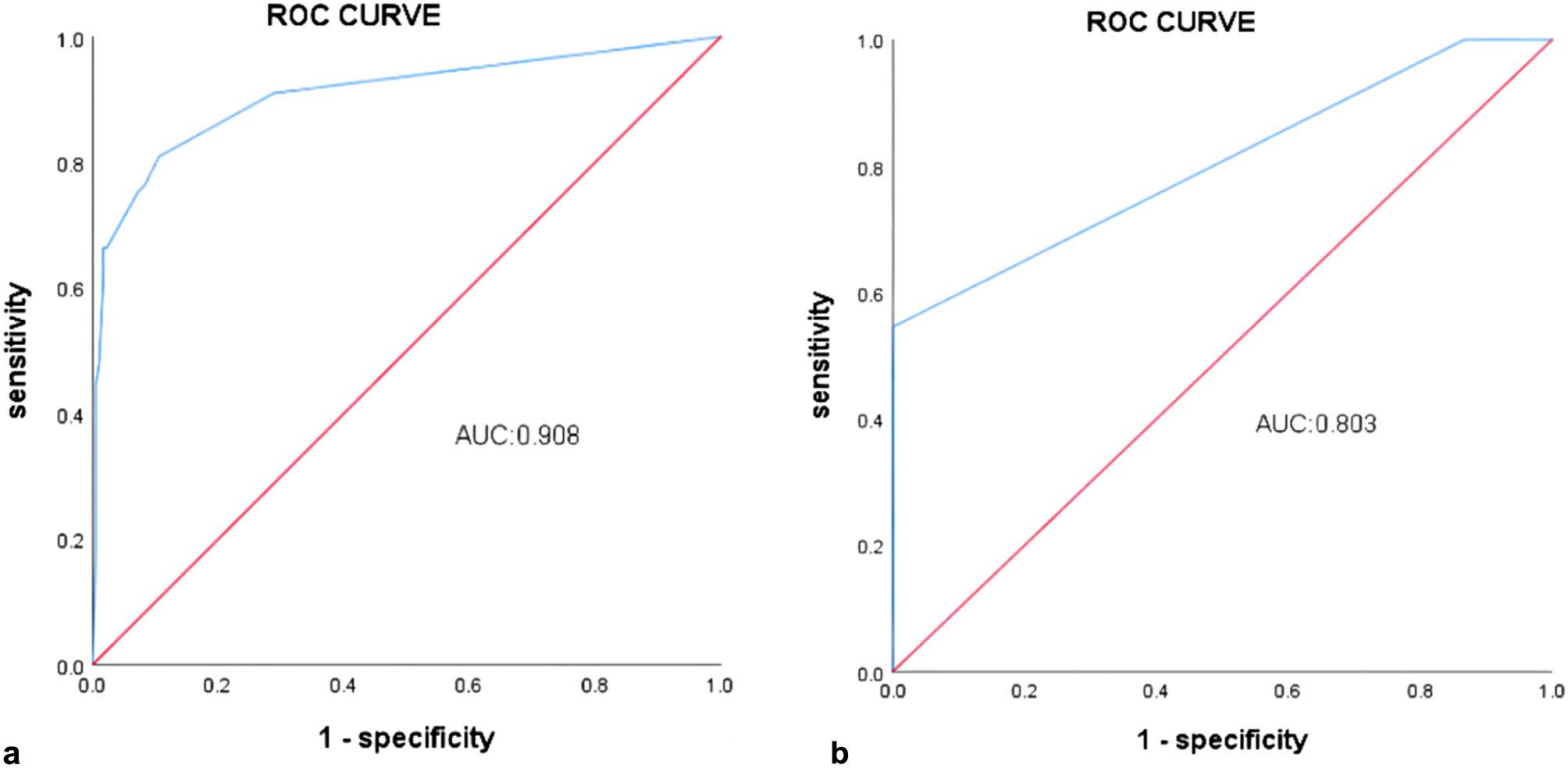
Receiver operating characteristic curve (ROC) for evaluating the discriminative performance of training cohort (**a**) and validation cohort (**b**).

The model’s discriminative ability was assessed using ROC curves. The AUC was 0.908 for the training cohort (Fig. 5a) and 0.803 for the validation cohort (Fig. 5b), indicating good predictive performance. The optimal cut-off values were 0.703 for the training cohort and 0.545 for the validation cohort, with corresponding sensitivities of 80.9% and 54.5%, and specificities of 89.4% and 100%.

**Figure 6:**
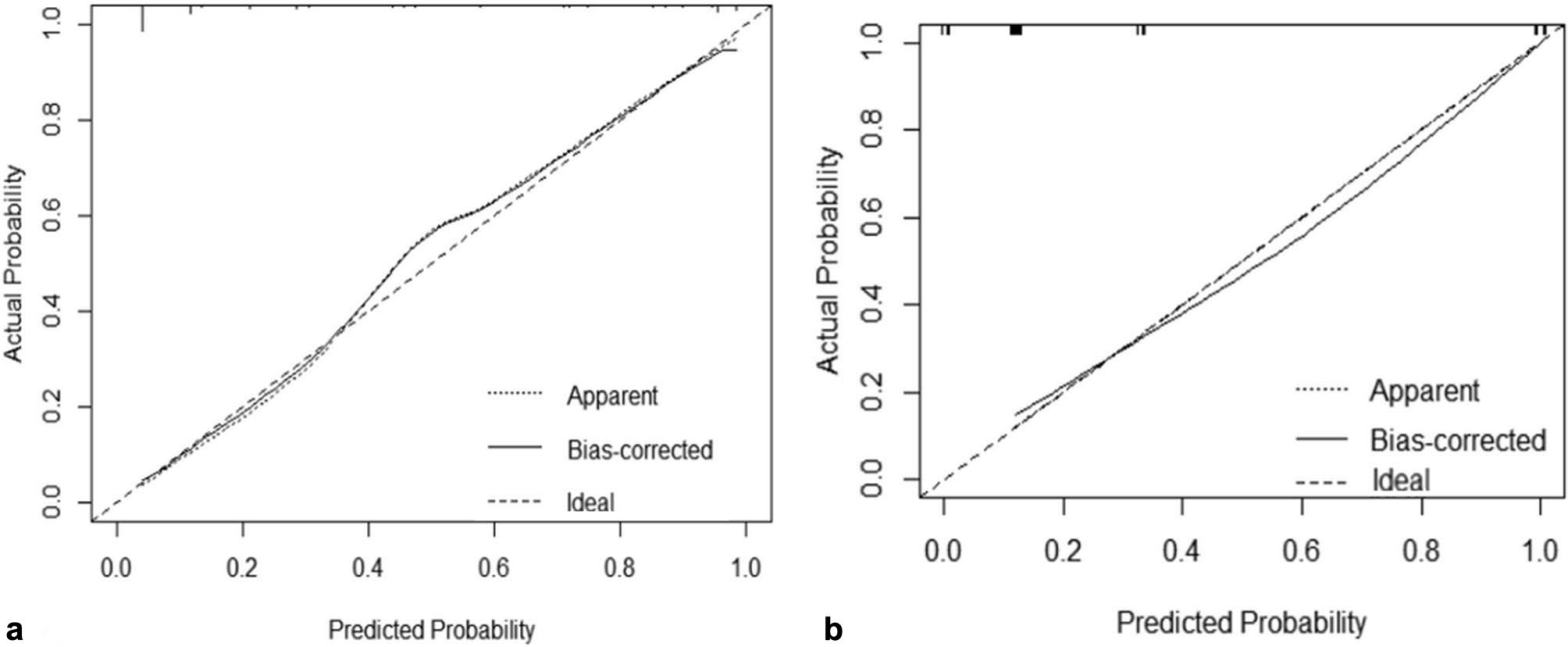
The calibration curve of the nomogram the in the training set (**a**) and validation set (**b**).

**Figure 7:**
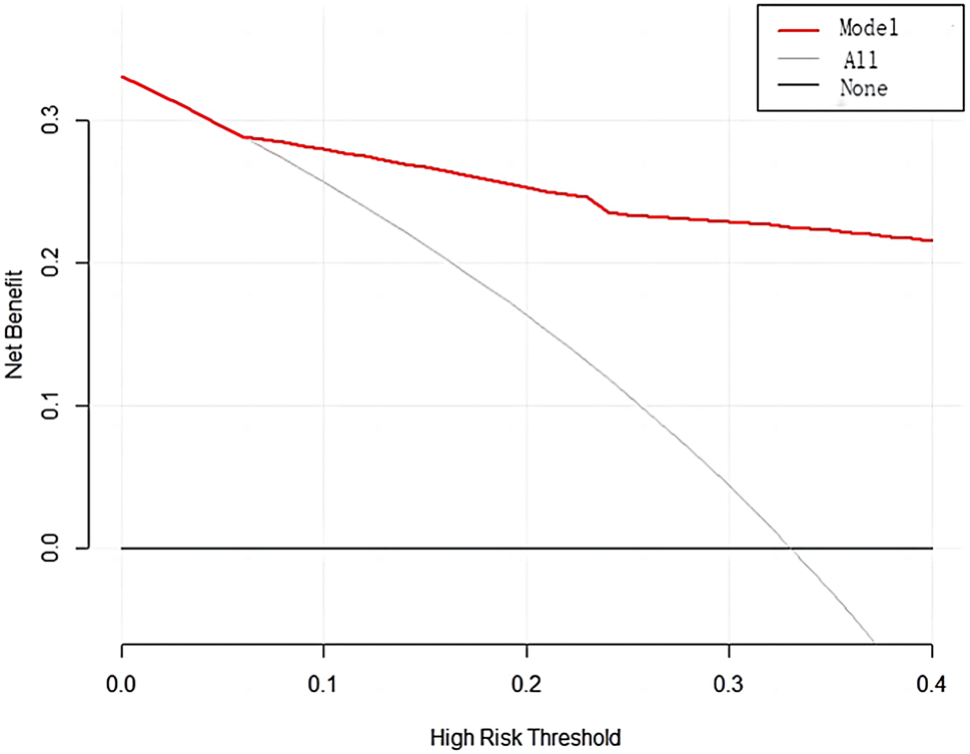
Decision curve analysis of nomogram model in a validation cohort.

Calibration of the model was performed, as depicted in Fig. 6a, b. The prediction curve closely matched the standard curve, with an average error less than 0.05, demonstrating accurate calibration. DCA, shown in Fig. 7, was conducted to evaluate the model’s clinical utility. The analysis indicated that using the nomogram for predicting invasive placenta probability is more beneficial than treating all or none of the patients when the threshold probability is greater than 8%.

## Discussion

Nomograms serve as simplified predictive models, presenting visual results in a graphical format. They are extensively employed in the prediction of tumorigenesis and prognosis for various cancers, including breast, gastric, and prostate cancers^18–20^. Prior research has primarily focused on the association between MRI signs and the occurrence of PAS^21–27^. In this study, we developed and validated a nomogram for predicting PAS in patients with placenta previa, incorporating clinical features and MRI signs. The nomogram, comprising the number of previous cesarean sections ≥ 2, placental bulge type-II, placenta covering scar, and placental protrusion sign, proved to be a practical and effective tool for clinical decision-making.

In our study, age and the number of previous abortions did not significantly differ between the noninvasive and invasive groups, aligning with Baughman’s findings^28^. The FIGO guideline suggests that, in comparison to cesarean sections, induced abortions have a minimal impact on the myometrium of the uterus, thereby posing a lower risk of invasive placenta. Our research identified that having two or more prior cesarean sections was an independent risk factor for PAS. This is attributed to the disruption of endometrial decidualization over uterine scarring areas, allowing villous trophoblasts to abnormally anchor deeply into the myometrium. This leads to uncontrolled invasion by extra-villous trophoblasts, a characteristic feature of PAS^29^.

While numerous MRI features of PAS have been extensively described in literature^21–27^, and many studies have demonstrated a strong correlation between MRI findings and intraoperative observations of invasive placentas^6,16,21^, certain MRI signs were excluded from our study. These include myometrial thinning, interrupted myometrium, heterogeneous placenta, abnormal uterine bulging, loss of the placental-myometrial interface, and percretism signs. The signs of interrupted myometrium and myometrial thinning, characterized by abrupt interruption or thinning of the myometrium to less than 1 mm at the site of focal placental bulging, often co-occur with placental bulging. Myometrial thinning, in particular, can lead to significant observational bias^30^. The heterogeneous placenta sign, indicating varied signal intensity within the placenta, is highly influenced by placental maturity and various other factors. Abnormal uterine bulging, manifested as widening of the lower uterine segment resembling an hourglass shape, lacks a precise definition in existing studies and is often confounded with focal placental bulge. The loss of the placental-myometrial interface becomes challenging to discern in cases of thin myometrium during late pregnancy. Percretism, indicating direct invasion into adjacent organs, has a notably low sensitivity. In our experience, the bridging vessels sign, often observed in type I PAS, can be difficult to distinguish from the sign of placental hypervascularity. As a result, we combined the bridging vessels sign with the placental hypervascularity sign for analysis in this study.

In this study, we focused on eight prominent and practical MRI signs, discovering that placental bulge type II, placenta covering scar, and placental protrusion were independent risk factors for invasive placenta. Jha et al. reported that the presence of a placental bulge, when combined with other indicators of invasive placenta, achieved a predictive ability of 100%^31^. Leyendecker similarly identified outward placental bulging as the most indicative sign^22^. Consistent with these findings, our study confirmed its independent risk factor status, with a sensitivity of 57.3% and a specificity of 98.3%. The sign of placenta covering scar, often associated with previous cesarean sections, demonstrated high sensitivity (83.10%) and specificity (75.00%) in our study. The placental protrusion sign, indicative of uncontrolled villous proliferation in the absence of a regular basal plate, showed a high specificity of 97.8%, although the sensitivity was only 44.90%. This aligns with Ueno’s findings, which associated this sign with invasive placenta praevia^32^. Our results reinforce the importance of these MRI signs as valuable predictors in the assessment of invasive placenta risk.

The model’s efficacy was validated in an external cohort, focusing on its discrimination, calibration, and clinical applicability. This validation offers a foundation for developing personalized treatment strategies for patients prior to surgery. The model demonstrated robust predictive accuracy, with an AUC of 0.908 in the training cohort and 0.803 in the validation cohort. These results affirm the model’s effectiveness, substantiated through external validation.

In 2021, Li developed and validated the first nomogram for PAS, based on a retrospective single-center study involving 118 patients in the training cohort and 65 in the validation cohort, drawn from different periods^11^. Our study encompasses a larger training cohort. A key distinction between our study and Li’s lies in the selection of MRI signs. Li’s study included signs like focal myometrial thinning less than 1 mm, focal myometrial interruption, intraplacental T2 dark bands, hemorrhage within the placenta, disorganized abnormal placental vascularity, abnormal uterine bulging, and loss of the double-line sign. We noted previously that the precise definition of uterine bulging is unclear and often confounded with placental bulging. Placental bulging is a frequent observation, typically coinciding with signs of myometrial thinning, interruption, and loss of the placental-myometrial interface. Therefore, we opted for Chen’s more intuitive and observer-stable classification of placental bulge^16^. This sign demonstrated the highest value in our nomogram. Additionally, the sensitivity of the newly introduced loss of double-line sign by Li was only 55.9%. Our nomogram achieved an AUC of 90.8%, surpassing the performance of Li's nomogram.

### Limitations

While our study successfully developed a high-accuracy nomogram model for PAS prediction, it is not without its shortcomings. The retrospective nature and relatively small sample size of the study may have introduced selection bias. Additionally, subjective biases in interpreting MRI signs could have affected the results. The stability and reliability of the model’s predictive performance also require further validation. To mitigate these biases and validate the effectiveness of the nomogram, additional research is necessary. Future research avenues include radiomics and advanced computational learning methods. Further studies, encompassing larger populations and multi-center collaborations, are essential to robustly validate and refine the prediction model.

## Conclusion

Utilizing a combination of clinical and MRI characteristics, we have established a highly accurate predictive model for invasive placenta in patients with placenta previa. Implementing this nomogram in clinical practice may enhance the preparation for and precision of treatment strategies, ultimately improving patient outcomes.

## Data Availability

The raw data supporting the conclusion of this article will be made available from the corresponding author upon request, without undue reservation.

## Abbreviations

CI: Confidence interval
DCA: Decision curve analysis
OR: Odds ratio
AUC: Area under the curve
PAS: Placenta accreta spectrum

## References

[1] Silver RM, Branch DW (2018). Placenta accreta spectrum. N. Engl. J. Med..

[2] Jauniaux E, Chantraine F, Silver RM, Langhoff-Roos J (2018). FIGO placenta accreta diagnosis and management expert consensus panel. FIGO consensus guidelines on placenta accreta spectrum disorders: Epidemiology. Int. J. Gynaecol. Obstet..

[3] Camuzcuoglu H, Toy H, Vural M, Yildiz F, Aydin H (2010). Internal iliac artery ligation for severe postpartum hemorrhage and severe hemorrhage after postpartum hysterectomy. J. Obstet. Gynaecol. Res..

[4] Carnevale FC, Kondo MM, de Oliveira Sousa W, Santos AB, da Motta Leal Filho JM, Moreira AM, Baroni RH, Francisco RP, Zugaib M (2011). Perioperative temporary occlusion of the internal iliac arteries as prophylaxis in cesarean section at risk of hemorrhage in placenta accreta. Cardiovasc. Intervent. Radiol..

[5] Oztas E, Ozler S, Ersoy AO, Ersoy E, Caglar AT, Uygur D, Yucel A, Ergin M, Danisman N (2016). Decreased placental and maternal serum TRAIL-R2 levels are associated with placenta accreta. Placenta.

[6] Bourgioti C, Zafeiropoulou K, Fotopoulos S, Nikolaidou ME, Antoniou A, Tzavara C, Moulopoulos LA (2018). MRI features predictive of invasive placenta with extrauterine spread in high-risk gravid patients: A prospective evaluation. AJR Am. J. Roentgenol..

[7] Tikkanen M, Paavonen J, Loukovaara M, Stefanovic V (2011). Antenatal diagnosis of placenta accreta leads to reduced blood loss. Acta Obstet. Gynecol. Scand..

[8] Warshak CR, Ramos GA, Eskander R, Benirschke K, Saenz CC, Kelly TF, Moore TR, Resnik R (2010). Effect of predelivery diagnosis in 99 consecutive cases of placenta accreta. Obstet. Gynecol..

[9] Ueno Y, Maeda T, Tanaka U, Tanimura K, Kitajima K, Suenaga Y, Takahashi S, Yamada H, Sugimura K (2016). Evaluation of interobserver variability and diagnostic performance of developed MRI-based radiological scoring system for invasive placenta previa. J. Magn. Reson. Imaging..

[10] Delli Pizzi A, Tavoletta A, Narciso R, Mastrodicasa D, Trebeschi S, Celentano C, Mastracchio J, Cianci R, Seccia B, Marrone L, Liberati M, Cotroneo AR, Caulo M, Basilico R (2019). Prenatal planning of placenta previa: Diagnostic accuracy of a novel MRI-based prediction model for placenta accreta spectrum (PAS) and clinical outcome. Abdom Radiol. NY..

[11] Li Q, Zhou H, Zhou K, He J, Shi Z, Wang Z, Dai Y, Hu Y (2021). Development and validation of a magnetic resonance imaging-based nomogram for predicting invasive forms of placental accreta spectrum disorders. J. Obstet. Gynaecol. Res..

[12] Riley RD, Ensor J, Snell KIE, Harrell FE, Martin GP, Reitsma JB, Moons KGM, Collins G, van Smeden M (2020). Calculating the sample size required for developing a clinical prediction model. BMJ.

[13] Wu S, Kocherginsky M, Hibbard JU (2005). Abnormal placentation: twenty-year analysis. Am. J. Obstet. Gynecol..

[14] Miller DA, Chollet JA, Goodwin TM (1997). Clinical risk factors for placenta previa-placenta accreta. Am. J. Obstet. Gynecol..

[15] Jha P, Pōder L, Bourgioti C, Bharwani N, Lewis S, Kamath A, Nougaret S, Soyer P, Weston M, Castillo RP, Kido A, Forstner R, Masselli G (2020). Society of Abdominal Radiology (SAR) and European Society of Urogenital Radiology (ESUR) joint consensus statement for MR imaging of placenta accreta spectrum disorders. Eur. Radiol..

[16] Chen X, Shan R, Zhao L, Song Q, Zuo C, Zhang X, Wang S, Shi H, Gao F, Qian T, Wang G, Limperopoulos C (2018). Invasive placenta previa: Placental bulge with distorted uterine outline and uterine serosal hypervascularity at 1.5T MRI–valuable features for differentiating placenta percreta from placenta accreta. Eur. Radiol..

[17] Jauniaux E, Ayres-de-Campos D, Langhoff-Roos J, Fox KA, Collins S (2019). FIGO placenta accreta diagnosis and management expert consensus panel. FIGO classification for the clinical diagnosis of placenta accreta spectrum disorders. Int. J. Gynaecol. Obstet..

[18] Deng J, Zhang D, Zhang W, Li J (2021). Construction and validation of new nomograms to predict risk and prognostic factors of breast cancer bone metastasis in asian females: A population-based retrospective study. Int. J. Gen. Med..

[19] Pan S, An W, Tan Y, Chen Q, Liu P, Xu H (2021). Prediction model of lymph node metastasis risk in elderly patients with early gastric cancer before endoscopic resection: A retrospective analysis based on international multicenter data. J Cancer..

[20] Clement C, Maurin C, Villeret J, Marcy M, Salem N, Brunelle S, Gravis G, Garcia S, Giusiano S, Bastide C, Rossi D, Bladou F, Walz J (2014). Head to head comparison of two currently used nomograms predicting the risk of side specific extra capsular extension to indicate nerve sparing during radical prostatectomy for treatment of prostate cancer. Prog. Urol..

[21] Stewart MJ, Richmond D, Mooney S, Esler S, Churilov L, Israelsohn N, Yang N (2021). Diagnostic utility of MRI features of placental adhesion disorder for abnormal placentation and massive postpartum hemorrhage. AJR Am. J. Roentgenol..

[22] Leyendecker JR, DuBose M, Hosseinzadeh K, Stone R, Gianini J, Childs DD, Snow AN, Mertz H (2012). MRI of pregnancy-related issues: abnormal placentation. AJR Am. J. Roentgenol..

[23] Jha P, Masselli G, Ohliger MA, Pōder L (2020). Nonfetal imaging during pregnancy: Placental disease. Radiol. Clin. North Am..

[24] Srisajjakul S, Prapaisilp P, Bangchokdee S (2014). MRI of placental adhesive disorder. Br. J. Radiol..

[25] Thiravit S, Lapatikarn S, Muangsomboon K, Suvannarerg V, Thiravit P, Korpraphong P (2017). MRI of placenta percreta: differentiation from other entities of placental adhesive disorder. Radiol. Med..

[26] Ishibashi H, Miyamoto M, Shinnmoto H, Murakami W, Soyama H, Nakatsuka M, Natsuyama T, Yoshida M, Takano M, Furuya K (2017). Cervical varicosities may predict placenta accreta in posterior placenta previa: A magnetic resonance imaging study. Arch. Gynecol. Obstet..

[27] Chu C, Zhao S, Ding M, Liu M, Zhang Y, Bao L, Wang D, Li W (2019). Combining clinical characteristics and specific magnetic resonance imaging features to predict placenta accreta. J. Comput. Assist. Tomogr..

[28] Baughman WC, Corteville JE, Shah RR (2008). Placenta accreta: Spectrum of US and MR imaging findings. Radiographics.

[29] Jauniaux E, Collins S, Burton GJ (2018). Placenta accreta spectrum: pathophysiology and evidence-based anatomy for prenatal ultrasound imaging. Am. J. Obstet. Gynecol..

[30] Alamo L, Anaye A, Rey J, Denys A, Bongartz G, Terraz S, Artemisia S, Meuli R, Schmidt S (2013). Detection of suspected placental invasion by MRI: Do the results depend on observer' experience?. Eur. J. Radiol..

[31] Jha P, Rabban J, Chen LM, Goldstein RB, Weinstein S, Morgan TA, Shum D, Hills N, Ohliger MA, Poder L (2019). Placenta accreta spectrum: value of placental bulge as a sign of myometrial invasion on MR imaging. Abdom. Radiol. NY..

[32] Ueno Y, Kitajima K, Kawakami F, Maeda T, Suenaga Y, Takahashi S, Matsuoka S, Tanimura K, Yamada H, Ohno Y, Sugimura K (2014). Novel MRI finding for diagnosis of invasive placenta praevia: evaluation of findings for 65 patients using clinical and histopathological correlations. Eur. Radiol..

